# Phospholipase Cγ2 is required for basal but not oestrogen deficiency–induced bone resorption

**DOI:** 10.1111/j.1365-2362.2011.02556.x

**Published:** 2012-01

**Authors:** Zsuzsanna Kertész, Dávid Győri, Szandra Körmendi, Tünde Fekete, Katalin Kis-Tóth, Zoltán Jakus, Georg Schett, Éva Rajnavölgyi, Csaba Dobó-Nagy, Attila Mócsai

**Affiliations:** * Department of Physiology, Semmelweis University School of MedicineBudapest, Hungary; † Independent Section of Radiology, Semmelweis UniversityBudapest, Hungary; ‡ Department of Immunology, Medical and Health Science Center, University of DebrecenDebrecen, Hungary; § Department of Internal Medicine 3, University of Erlangen-NurembergErlangen, Germany

**Keywords:** Gene expression, knockout mice, osteoclasts, osteoporosis, phospholipase Cγ2, signalling

## Abstract

**Background:**

Osteoclasts play a critical role in bone resorption under basal conditions, but they also contribute to pathological bone loss during diseases including postmenopausal osteoporosis. Phospholipase Cγ2 (PLCγ2) is an important signalling molecule in diverse haematopoietic lineages. Here, we tested the role of PLCγ2 in basal and ovariectomy-induced bone resorption, as well as in *in vitro* osteoclast cultures using PLCγ2-deficient (PLCγ2^−/−^) mice.

**Materials and methods:**

The trabecular architecture of long bone metaphyses was tested by micro-CT and histomorphometric analyses. Postmenopausal osteoporosis was modelled by surgical ovariectomy. Osteoclast development and function, gene expression and PLCγ2 phosphorylation were tested on *in vitro* osteoclast and macrophage cultures.

**Results:**

PLCγ2^−/−^ mice had significantly higher trabecular bone mass under basal conditions than wild-type mice. PLCγ2 was required for *in vitro* development and resorptive function of osteoclasts, but not for upregulation of osteoclast-specific gene expression. PLCγ2 was phosphorylated in a Src-family-dependent manner upon macrophage adhesion but not upon stimulation by M-CSF or RANKL. Surprisingly, ovariectomy-induced bone resorption in PLCγ2^−/−^ mice was similar to, or even more robust than, that in wild-type animals.

**Conclusions:**

Our results indicate that PLCγ2 participates in bone resorption under basal conditions, likely because of its role in adhesion receptor signalling during osteoclast development. In contrast, PLCγ2 does not appear to play a major role in ovariectomy-induced bone loss. These results suggest that basal and oestrogen deficiency–induced bone resorption utilizes different signalling pathways and that PLCγ2 may not be a suitable therapeutic target in postmenopausal osteoporosis.

## Introduction

While osteoclasts are important for normal bone turnover, they also contribute to pathological bone loss during osteoporosis, rheumatoid arthritis or osteolytic bone metastases [1–3]. However, it is incompletely understood how osteoclasts contribute to normal and pathological bone resorption and whether they utilize similar intracellular signalling machineries during the two processes.

Osteoclasts are highly specialized phagocytic cells of haematopoietic origin [4]. They develop by an initial macrophage-like differentiation, followed by reprogramming to the osteoclast lineage and fusion of preosteoclasts to mature multinucleated osteoclasts [1,4]. These processes are directed by the M-CSF and the osteoblast-derived RANK ligand (RANKL) cytokines.

A number of recent studies have indicated similar components of osteoclast biology and immune mechanisms, leading to the emergence of the new field of osteoimmunology [5]. Those similarities include activation by closely related cytokines [5–7], shared use of transcription factors [5,8,9] and the role of immunoreceptor-like signalling pathways (such as Syk activation by immunoreceptor-associated adapters) in osteoclast development [10–14]. The similarity between bone and immune cells is further supported by the similar components used by neutrophil, macrophage and osteoclast signalling and *in vivo* inflammatory processes [15–19].

Phospholipase Cγ (PLCγ) proteins link tyrosine kinase-coupled receptors to Ca^2+^ signalling and PKC activation [20]. While the PLCγ1 isoform is ubiquitously expressed and is required for embryonic development [21,22], PLCγ2 is primarily present in haematopoietic lineage cells and its absence triggers defects in haematopoietic lineage cells [23–28]. Most of those phenotypes are also shared with Syk^−/−^ mice or mice with genetic defects of immunoreceptor signalling molecules [15,19,29,30].

PLCγ2 is also activated downstream of the immunoreceptor signalling adapters DAP12 and the Fc-receptor γ-chain (FcRγ) in osteoclast precursors while the downstream activation of the NFATc1 transcription factor is mediated by Ca^2+^ signalling through tyrosine phosphorylation pathways [11,31]. The overall similarity between immunoreceptor and PLCγ2-mediated signalling pathways suggests a possible role for PLCγ2 in osteoclast biology.

The above results prompted us to test the role of PLCγ2 in osteoclast development and function, as well as in *in vivo* bone homeostasis under normal and pathological conditions. Our results indicate that PLCγ2 plays an important role in basal bone resorption, likely due to its role in later phases of osteoclast development. Surprisingly, however, PLCγ2 does not play a major role in ovariectomy-induced bone loss.

## Materials and methods

### Animals

Heterozygous mice carrying a deleted allele of the PLCγ2-encoding gene (*Plcg2*^tm1Jni^, referred to as PLCγ2^−^) [23] were obtained from James N. Ihle (St. Jude Children’s Research Hospital, Memphis, TN, USA) and has been backcrossed to the C57BL/6 genetic background for more than 10 generations. Because of the limited fertility of homozygous PLCγ2^−/−^ mice, the mutation was maintained in heterozygous form as described [26].

For *in vivo* experiments, PLCγ2^+/+^ or PLCγ2^+/−^ mice of identical age and sex (mostly littermates) from the same colony were used as controls. For *in vitro* experiments, either PLCγ2-sufficient mice from the PLCγ2 breeding colony or C57BL/6 mice purchased from the Hungarian National Institute of Oncology (Budapest, Hungary) were used as controls. Because of the limited availability of PLCγ2^−/−^ animals, some of the *in vitro* experiments were performed on cells from PLCγ2^−/−^ (and appropriate control) bone marrow chimeras generated and tested as described [26]. No difference between the different sources of mice or bone marrow cells has been observed (not shown).

Mice were kept in individually sterile ventilated cages (Tecniplast, Buguggiate, Italy) in a conventional facility. All animal experiments were approved by the Semmelweis University Animal Experimentation Review Board.

### Ovariectomy

To test oestrogen deficiency–induced bone loss, wild-type and PLCγ2^−/−^ females at 8 weeks of age were anesthetized with ketamine and medetomidine and subjected to surgical ovariectomy or sham operation. Six weeks after the operation, the mice were sacrificed and their femurs or tibias were analysed.

### Micro-CT and histomorphometry

Bone architecture under basal conditions was tested on age-matched wild-type and PLCγ2^−/−^ male mice at 8–10 weeks of age. Ovariectomy-induced bone loss was tested at 14 weeks of age on wild-type and PLCγ2^−/−^ females.

Micro-CT studies were performed on the distal metaphysis of the femurs stored in PBS containing 0·1% Na-azide. Samples were scanned on a SkyScan 1172 (SkyScan, Kontich, Belgium) micro-CT apparatus using a 50 kV and 200 μA X-ray source with 0·5-mm aluminium filter, and a rotation step of 0·5° with frame averaging turned on, resulting in an isometric voxel size of 4·5 μm. Three-dimensional images were reconstituted and analysed using the NRecon and CT-Analyser software (both from SkyScan). For quantitative analysis, 400 horizontal sections starting 50 sections above the distal growth plate were selected, and the boundaries of trabecular area were selected manually a few voxels away from the endocortical surface [32]. The density threshold for bone tissue was set manually by an experienced investigator. For graphical presentation, the two-dimensional representation of a horizontal section 250 sections above the distal growth plate, as well as the three-dimensional reconstitution of an axial cylinder of 700 μm diameter, expanding from 150 to 450 sections above the distal growth plate has been prepared.

Histomorphometry studies were performed on the proximal metaphysis of the tibias. After sacrificing the mice, the bones were placed in 70% ethanol, then fixed overnight in 4% formalin and embedded undecalcified in methylmetacrylate (Technovit; Heraeus Kulzer, Wehrheim, Germany). After polymerization, 3- to 4-μm sections were cut with a Jung micrometer (Jung, Heidelberg, Germany) and deplastinated in methoxymethylmetacrylate (Merck, Darmstadt, Germany). Sections were stained with von Kossa and Goldner stains. Bone histomorphometry was performed using a microscope (Nikon, Tokyo, Japan) equipped with a video camera and digital analysis system (OsteoMeasure; OsteoMetrics, Decatur, GA, USA). Histomorphometry parameters were measured according to international standards [33] as described [34].

### *In vitro* cultures, resorption assays and flow cytometry

Suspensions of bone marrow cells were cultured for 48 h in α-MEM (Invitrogen, Carlsbad, CA, USA) in the presence of 10 ng/mL recombinant mouse M-CSF (Peprotech, Rocky Hill, NJ, USA). Nonadherent cells (osteoclast/macrophage precursors) were then plated at 0·5 million per cm^2^ and cultured in the presence of 20 or 50 ng/mL mouse M-CSF or mouse RANKL (Peprotech) with media changes and replacement of the cytokines every 2–3 days. Cellular morphology and tartrate-resistant acid phosphatase (TRAP) expression were determined after 3–5 days of culture in 24-well tissue culture-treated plates using a commercial TRAP staining kit (Sigma, St. Louis, MO, USA). For *in vitro* resorption assays, osteoclast precursors were plated on BD BioCoat Osteologic slides (BD Biosciences, Bedford, MA, USA), cultured in the presence of M-CSF and RANKL for 10–14 days and processed according to the manufacturer’s instructions. Cultures were observed and imaged using a Leica Microsystems (Wetzlar, Germany) DMI6000B inverted microscope. The number of osteoclasts (i.e. TRAP-positive cells with 3 or more nuclei) was counted manually, while the percentage of resorbed area was determined using the ImageJ software (NIH, Bethesda, MD, USA).

Macrophages were generated by culturing osteoclast/macrophage precursors in the presence of M-CSF but not RANKL. M-CSF was supplied in the form of purified protein (parallel macrophage and osteoclast studies) or as a 10% conditioned medium from CMG14-12 cells [35] (biochemical studies). Expression of the F4/80 macrophage differentiation antigen was tested as described [19].

### Analysis of gene expression

Osteoclast-specific gene expression was tested using quantitative real-time PCR analysis [36] from wild-type or PLCγ2^−/−^ cultures generated in the indicated periods of time using the indicated cytokine concentrations. Total RNA was then isolated from the cells with Trizol reagent (Invitrogen). Reverse transcription was performed at 37 °C for 120 min from 100 ng total RNA using the High Capacity cDNA Archive Kit (Applied Biosystems, Foster City, CA, USA). Quantitative real-time PCRs were performed in triplicates with a control reaction containing no reverse transcriptase on an ABI PRISM 7900 (Applied Biosystems) equipment with 40 cycles at 94 °C for 12 s and 60 °C for 60 s using Applied Biosystems Taqman Gene Expression Assay kits. We tested the expression of the mouse *Acp5* (TRAP; Taqman Mm00475698_m1), *Calcr* (Calcitonin receptor; Mm00432271_m1), *Ctsk* (cathepsin K; Mm00484039_m1), *Fos* (c-Fos; Mm00487425_m1), *Nfatc1* (NFATc1; Mm00479445_m1), *Oscar* (OSCAR; Mm00558665_m1) and *Tm7sf4* (DC-STAMP; Mm04209235_m1) genes and normalized it to the expression of the housekeeping gene *Gapdh* (GAPDH; Mm99999915_g1). The comparative C_t_ method was used to quantify transcripts.

### Biochemical and signalling studies

PLCγ2 expression was determined from Triton X-100-soluble whole osteoclast or macrophage lysates as described [26].

For signalling studies, macrophages were cultured for 5–8 days in bacterial dishes, suspended with 5 mM EDTA and serum starved for 6 h. When indicated, the cells were then incubated with 10 μM PP2 (EMD Biosciences, Darmstadt, Germany) for 8 min. The cells were stimulated with 50 ng/mL M-CSF or 50 ng/mL RANKL in suspension or were plated on 6-cm tissue culture-treated dishes. The reaction was stopped after 30 min at 37 °C, and cell lysates were prepared as described [26]. PLCγ2 was precipitated using the Q-20 PLCγ2 antibody (Santa Cruz Biotechnology, Santa Cruz, CA, USA) and captured using a 1 : 1 mixture of Protein A Sepharose (Zymed, South San Francisco, CA, USA) and Protein G Agarose (Invitrogen). Whole-cell lysates or PLCγ2 immunoprecipitates were immunoblotted with phosphorylation-specific antibodies (from Cell Signaling Technology, Danvers, MA) against PLCγ2 (pTyr 759; #3874), ERK (#9101) or the p38 MAP kinase (#9211); nonphospho-specific antibodies against PLCγ2 (Q-20; Santa Cruz), ERK1/2 (combination of C-16 (ERK1) and C-14 (ERK2) from Santa Cruz), p38 MAP kinase (C-20; Santa Cruz), IκBα (#9242; Cell Signaling) or β-actin (AC-74; Sigma); or antibodies against phosphotyrosine (clone 4G10; Millipore, Billerica, MA, USA). Signal intensity was developed using secondary antibodies and ECL reagents from GE Healthcare (Chalfont St. Giles, UK).

### Statistical analysis

Experiments were performed at the indicated times with comparable results. Statistical analyses were performed using Student’s unpaired two-population *t*-test with unequal variance or by two-way anova. Analysis of the interaction between the effects of genotypes and surgical treatments was performed using Tukey’s post hoc test. *P* values below 0·05 were considered statistically significant.

## Results

### micro-CT and histomorphometric analysis of wild-type and PLCγ2^−/−^ animals

**Figure 1:**
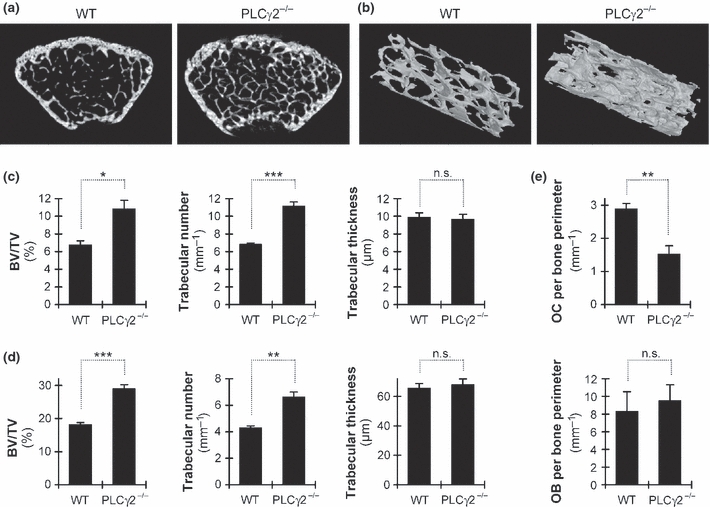
Micro-CT and histomorphometric analysis of intact PLCγ2^−/−^ mice. (a–b) Representative micro-CT sections (a) and three-dimensional reconstitution (b) of the trabecular area of the distal femoral metaphysis of age-matched wild-type (WT) and PLCγ2^−/−^ male mice. Distal regions are shown to the lower right in panel b. (c,d) Quantitative micro-CT (c) and histomorphometric (d) analysis of the trabecular bone architecture. (e) Histomorphometric analysis of the number of osteoclasts (OC) or osteoblasts (OB) attached to the trabecular bone surface. Data were obtained from five (a–c) or four (d,e) mice per group at 8–10 weeks of age. The analyses were performed on the distal metaphysis of the femurs (a–c) or the proximal metaphysis of the tibias (d,e). Error bars represent SEM. *P < 0·05; **P < 0·01; ***P < 0·002; n.s., not significant; BV/TV, per cent bone volume (bone volume/total volume).

We first analysed the composition of trabecular bone of wild-type and PLCγ2^−/−^ male mice using micro-CT analysis of the distal metaphysis of the femurs. Significantly more trabeculae were visible in PLCγ2^−/−^ animals than in the wild-type mice both in raw micro-CT slices (Fig. 1a) and in three-dimensional reconstitution images of an axial cylindrical region (Fig. 1b). Quantification of the entire three-dimensional reconstitution image (Fig. 1c) revealed a significant increase in the per cent bone volume (BV/TV) of PLCγ2^−/−^ animals (*P* = 0·011; *n* = 5), which was primarily because of increased trabecular number rather than increased thickness of the individual trabeculae (Fig. 1c).

We also performed histomorphometric analysis of the trabecular bone of the proximal tibia of male mice. Those studies confirmed an increased relative bone volume (BV/TV; *P* = 0·0012; *n* = 4) and trabecular number, but not trabecular thickness is PLCγ2^−/−^ animals (Fig. 1d). In addition, a significantly lower number of osteoclasts was seen in PLCγ2^−/−^ bones while the number of osteoblasts was not affected (Fig. 1e). Taken together, PLCγ2^−/−^ animals have increased trabecular bone volume likely due to an osteoclast defect.

### PLCγ2 is required for *in vitro* osteoclast development

**Figure 2:**
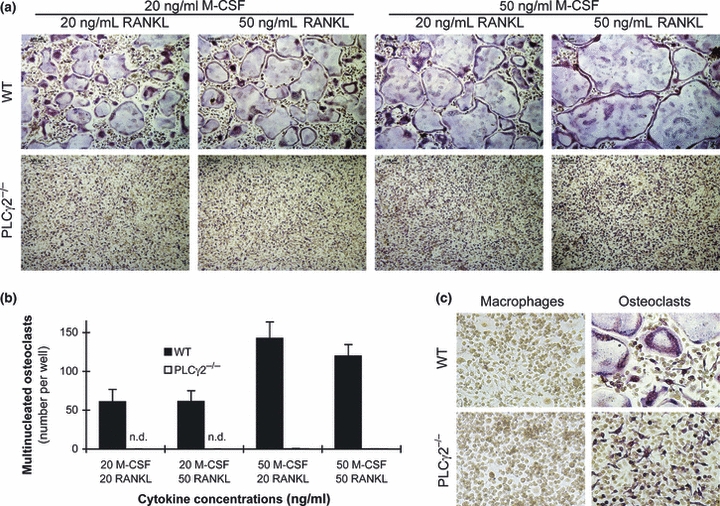
PLCγ2 is required for *in vitro* osteoclast development. (a) Representative TRAP-stained images of wild-type (WT) and PLCγ2^−/−^ bone marrow cells cultured in the presence of the indicated concentrations of recombinant murine M-CSF and RANKL for 4 days. (b) Number of multinucleated osteoclasts (TRAP-positive cells with 3 or more nuclei) in cultures of WT and PLCγ2^−/−^ bone marrow cells treated with the indicated concentrations of M-CSF and RANKL for 4 days. (c) Enlarged view of TRAP-stained osteoclast and macrophage cultures generated in the presence of 20 ng/mL M-CSF with (osteoclasts) or without (macrophages) 20 ng/mL RANKL for 4 days. Results were obtained from 15 (a,b) or 7 (c) independent experiments per group. Error bars represent SEM. n. d., not detected.

To test the role of PLCγ2 in osteoclasts, we have cultured wild-type and PLCγ2^−/−^ bone marrow cells under osteoclastogenic conditions *in vitro*. As shown in the TRAP-stained images in Fig. 2a and their quantification in Fig. 2b, 20 ng/mL M-CSF and 20 ng/mL RANKL induced significant osteoclast development from wild-type but not from PLCγ2^−/−^ bone marrow cells. This defect could not be overcome by increasing the concentration of M-CSF, RANKL or both to 50 ng/mL (Figs 2a,b). However, both wild-type and PLCγ2^−/−^ cultures consistently stained positive for TRAP (Fig. 2c), and the percentage of TRAP-positive cells among all mononuclear cells was very similar in the two genotypes (30-35% at 20 ng/mL M-CSF + 20 ng/mL RANKL and 40–45% at 50 ng/mL M-CSF and 50 ng/mL RANKL, irrespective of the genotype of the cells). Taken together, PLCγ2 is required for the *in vitro* development of mature multinucleated osteoclasts in the presence of M-CSF and RANKL but likely not for the initial steps of preosteoclast differentiation.

### PLCγ2 is required for *in vitro* bone resorption

**Figure 3:**
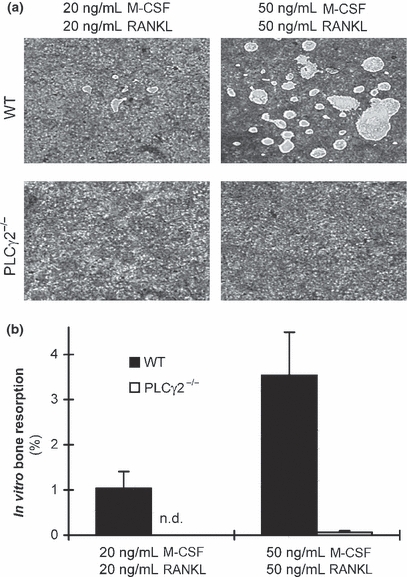
PLCγ2 is required for *in vitro* resorptive activity of osteoclasts. (a) Representative images of resorption of an artificial hydroxyapatite layer in wild-type (WT) and PLCγ2^−/−^ osteoclast cultures. Bone marrow cells were cultured on BD BioCoat Osteologic Plates in the presence of the indicated concentrations of M-CSF and RANKL for 14 days. (b) Quantification of the *in vitro* resorptive activity of WT and PLCγ2^−/−^ osteoclast cultures treated with the indicated concentrations of cytokines. Results were obtained from 3 to 7 independent experiments per group. Error bars represent SEM. n.d., not detected.

We next tested the effect of PLCγ2 deficiency on osteoclast-mediated bone resorption by culturing bone marrow cells on an artificial hydroxyapatite layer. As shown in Fig. 3, wild-type cells cultured in the presence of 20 ng/mL M-CSF and 20 ng/mL RANKL had a moderate resorptive capacity that was strongly increased by increasing the concentration of both cytokines to 50 ng/mL. In contrast, practically, no resorption could be observed in PLCγ2^−/−^ cultures at either cytokine concentration. Therefore, PLCγ2 is also required for osteoclast-mediated bone resorption, likely reflecting the previously mentioned osteoclast developmental defect (Fig. 2).

### PLCγ2 is not required for macrophage differentiation or expression of osteoclast-specific genes

**Figure 4:**
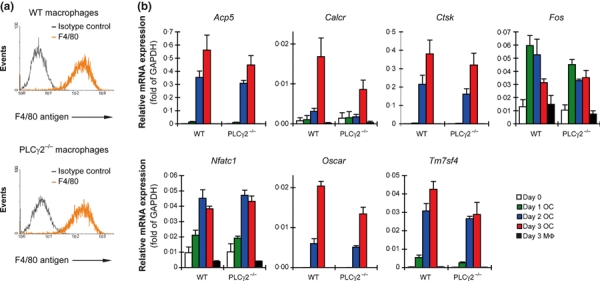
Normal macrophage development and osteoclast-specific gene expression. (a) Flow cytometric analysis of the expression of the F4/80 macrophage differentiation marker on wild-type (WT) and PLCγ2^−/−^ macrophages generated by culturing bone marrow cells in the presence of recombinant murine M-CSF. Curves of isotype control-stained cells show non-specific labelling. (b) Analysis of the expression of osteoclast-specific genes in WT and PLCγ2^−/−^ osteoclast (OC) and macrophage (MΦ) cultures. WT and PLCγ2^−/−^ bone marrow cells were cultured in the presence of 50 ng/mL with (OC) or without (MΦ) 50 ng/mL RANKL for the indicated period of time, and then the expression of the *Acp5* (TRAP), *Calcr* (Calcitonin receptor), *Ctsk* (Cathepsin K), *Fos* (c-Fos), *Nfatc1* (NFATc1), *Oscar* (OSCAR) and *Tm7sf4* (DC-STAMP) genes was determined using quantitative RT-PCR. The results shown were obtained from 3 to 6 independent experiments per group. Error bars represent SEM.

Our next aim was to address whether PLCγ2 is involved in an earlier or a later phase of osteoclast differentiation. Because we were able to obtain normal numbers of apparently normal macrophages from PLCγ2^−/−^ bone marrow cells (Fig. 2c and data not shown) and those macrophages expressed normal levels of the macrophage differentiation marker F4/80 (Fig. 4a), it is unlikely that PLCγ2 is required for the first steps of general myeloid cell differentiation.

We next tested the time course of osteoclast-specific gene expression in *in vitro* cultures by quantitative RT-PCR. As shown in Fig. 4b, the expression of the *Acp5* (encoding for TRAP), *Calcr* (calcitonin receptor), *Ctsk* (cathepsin K), *Fos* (c-Fos), *Nfatc1* (NFATc1), *Oscar* (OSCAR) and *Tm7sf4* (DC-STAMP) genes was strongly increased during osteoclast differentiation, but none of these genes showed increased expression in parallel macrophage samples. The genetic deficiency of PLCγ2 did not induce any major reduction in osteoclast-specific gene expression, although some partial decrease in expression could be observed, particularly in the case of *Calcr* (Fig. 4b). Most importantly, the expressions of the genes encoding for the early maturation marker TRAP (*Acp5*), the most plausible PLCγ2 effector NFATc1 (*Nfatc1*) [11,31] and of DC-STAMP (*Tm7sf4*), a critical player of the preosteoclast fusion machinery [37,38], were all upregulated normally in PLCγ2^−/−^ cultures (Fig. 4b). These results indicate that PLCγ2 is mostly dispensable for initiation of osteoclast-specific gene expression.

### Biochemical characterization of the PLCγ2-mediated osteoclast signalling pathway

**Figure 5:**
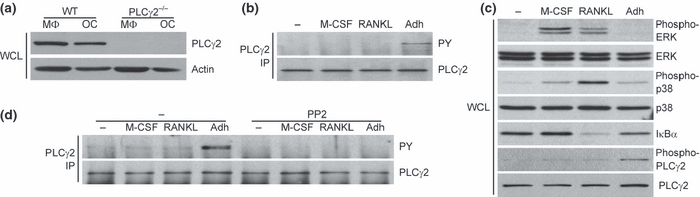
Cellular adhesion triggers PLCγ2 phosphorylation. (a) Expression of PLCγ2 in macrophages (MΦ) and osteoclasts (OC). Wild-type (WT) and PLCγ2^−/−^ bone marrow cells were cultured in the presence of 50 ng/mL M-CSF with (OC) or without (MΦ) 50 ng/mL RANKL for 4 days, followed by preparation of whole-cell lysates (WCL) and immunoblotting for PLCγ2 and β-actin. B-C, Stimulus-induced phosphorylation of PLCγ2. Wild-type macrophages were treated with 50 ng/mL M-CSF, 50 ng/mL RANKL or kept unstimulated in suspension, or they were plated on tissue culture-treated plastic dishes (Adh). After 30 min of incubation, cell was lysed and processed for immunoprecipitation (IP) of PLCγ2 followed by immunoblotting using anti-phosphotyrosine (PY) antibodies, (b) or WCL were immunoblotted using phosphorylation-specific antibodies against ERK, the p38 MAP kinase (p38) and PLCγ2 or nonphospho-specific antibodies against IκBα (c). Immunoblotting for ERK, p38 and PLCγ2 served as loading control. (d) Role of Src-family kinases in PLCγ2 phosphorylation. Wild-type macrophages were pretreated in the presence or absence of 10 μM PP2 and then stimulated and their PLCγ2 phosphorylation tested as in panel b. Results shown represent 3–5 independent experiments with similar results.

Next, we aimed at the biochemical characterization of PLCγ2 activation in osteoclasts. We first tested the presence of PLCγ2 in parallel macrophage and osteoclast cultures and found that PLCγ2 was expressed at comparable levels in wild-type macrophages and osteoclasts but, as expected, not in PLCγ2^−/−^ cells (Fig. 5a).

Osteoclast development is triggered by three major extracellular signals: M-CSF, RANKL and adhesive interactions with the environment (e.g. with tissue culture plastic surface). We next tested which of these three signals trigger PLCγ2 activation, using wild-type macrophages stimulated with M-CSF or RANKL in suspension (which was required to avoid parallel engagement of adhesion receptors), or plated on a tissue culture plastic surface. Both an immunoprecipitation approach followed by immunoblotting with anti-phosphotyrosine antibodies (Fig. 5b) and a direct immunoblotting using phospho-specific PLCγ2 antibodies (Fig. 5c) revealed PLCγ2 phosphorylation upon adhesion of macrophages but not upon M-CSF or RANKL stimulation in suspension. Additional attempts with M-CSF or RANKL stimulation for various periods of time or using various cytokine concentrations ranging from 10 to 100 ng/mL did not reveal a consistent PLCγ2 phosphorylation in suspension either (not shown). On the other hand, M-CSF-induced ERK phosphorylation and RANKL-induced p38 MAP kinase phosphorylation and NFκB activation (degradation of IκBα) could readily be observed under these conditions (Fig. 5c), indicating intact basic M-CSF and RANKL signalling in suspension. Therefore, PLCγ2 appears to be activated by adhesive interactions rather than by stimulation with M-CSF or RANKL cytokines.

We have also tested the role of Src-family kinases in PLCγ2 phosphorylation. As shown in Fig. 5d, pretreatment of macrophages with the Src-family inhibitor PP2 completely abrogated the PLCγ2 phosphorylation response, indicating that the adhesion-induced PLCγ2 activation requires members of the Src kinase family.

### PLCγ2^−/−^ mice show normal ovariectomy-induced bone resorption

**Figure 6:**
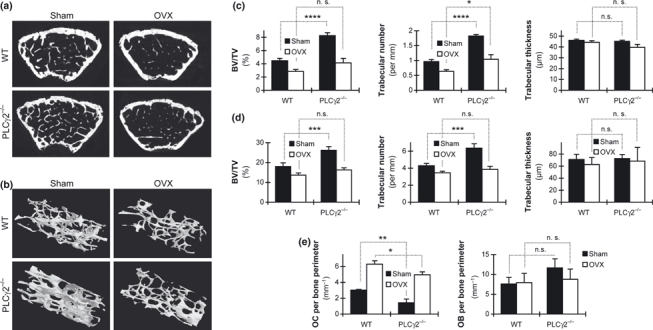
PLCγ2 is not required for ovariectomy-induced bone resorption. (a,b) Representative micro-CT sections (a) and three-dimensional reconstitution (b) of the trabecular area of the distal metaphysis of the femur of wild-type (WT) and PLCγ2^−/−^ female mice subjected to surgical ovariectomy (OVX) or a sham operation. Distal regions are shown to the lower right in panel b. (c,d) Quantitative micro-CT (c) or histomorphometric (d) analysis of the trabecular bone architecture of ovariectomized or sham-operated mice of the indicated genotypes. (e) Histomorphometric analysis of the number of osteoclasts (OC) or osteoblasts (OB) attached to the trabecular bone surface. Data were obtained from 7 (WT) or 4 (PLCγ2^−/−^) mice per group (a–c) or from three mice per group (d,e). Surgical operation was performed at 8 weeks of age followed by an additional 6 weeks before the mice were sacrificed and their bones were removed for analysis. Error bars represent SEM of the indicated number of animals. *P < 0·05; **P < 0·01; ***P < 0·002; ****P < 0·0004; n.s., not significant; BV/TV, per cent bone volume (bone volume/total volume).

Because osteoclast-mediated bone resorption contributes to postmenopausal osteoporosis [39], we hypothesized that PLCγ2 may also play a role in oestrogen deficiency–induced bone loss. That possibility was tested by subjecting wild-type and PLCγ2^−/−^ animals to surgical ovariectomy, followed by micro-CT and histomorphometric analysis 6 weeks later. Representative raw micro-CT sections (Fig. 6a), three-dimensional reconstitution images (Fig. 6b) and quantitative micro-CT analyses (Fig. 6c) indicated that, similar to intact male animals (Fig. 1), sham-operated PLCγ2^−/−^ females also had increased trabecular bone density, which was reflected in a nearly twofold increase in relative bone volume (BV/TV; *P* = 0·00031; *n* = 7 (wild type) vs. 4 (PLCγ2^−/−^)). As expected, surgical ovariectomy led to a significant reduction in the per cent bone volume (BV/TV) of wild-type mice (*P* = 0·025; *n* = 7). Contrary to our expectations, however, the per cent bone volume of PLCγ2^−/−^ animals was also significantly reduced (*P* = 0·00023; *n* = 4) and that reduction was even higher in PLCγ2^−/−^ mice than in wild-type animals both in terms of absolute reduction in BV/TV values (4·1 vs. 1·6 percentage points, respectively) and in percentage of the BV/TV values of the sham-operated control animals (50% vs. 36%, respectively). The difference of the effect of ovariectomy on wild-type and PLCγ2^−/−^ animals (interaction of the genotypes and surgical procedures) proved to be statistically significant (*P* = 0·0090). Importantly, while the BV/TV values of sham-operated wild-type and PLCγ2^−/−^ animals were statistically highly significant (*P* = 0·00025), there was no significant difference between the two genotypes after the ovariectomy procedure (*P* = 0·25; *n* = 7 (wild type) vs. 4 (PLCγ2^−/−^)). Similar differences could be observed in the trabecular numbers, whereas the trabecular thickness remained unaffected by the different genotypes and surgical procedures (Fig. 6c).

The above-mentioned findings were also confirmed by histomorphometric analysis of ovariectomy-induced bone loss in the proximal tibia. As shown in Fig. 6d, that analysis confirmed the increased per cent bone volume (BV/TV) in sham-operated PLCγ2^−/−^ animals (*P* = 0·0010; *n* = 3) and a reduction in per cent bone volume in ovariectomized wild-type mice (*P* = 0·038; *n* = 3). Importantly, the ovariectomy procedure induced a significantly more pronounced reduction in per cent bone volume in PLCγ2^−/−^ mice than in wild-type animals, both in terms of absolute reduction in BV/TV values (9·9 vs. 4·3 percentage points, respectively) and in per cent of the BV/TV values of the sham-operated control animals (38% vs. 24%, respectively). The difference of the effect of ovariectomy on wild-type and PLCγ2^−/−^ animals (interaction of the genotypes and surgical procedures) proved again to be statistically significant (*P* = 0·013). Similar to the micro-CT data (Fig. 6a), while the BV/TV values of sham-operated wild-type and PLCγ2^−/−^ animals were statistically highly significant (*P* = 0·0010), that difference faded away after the ovariectomy procedure (*P* = 0·24; *n* = 3). A similar picture was seen during the analysis of trabecular numbers, whereas the trabecular thickness remained unaffected by the different genotypes and surgical procedures (Fig. 6d).

Additional studies testing the number of osteoclasts and osteoblasts attached to the bone surface indicated that although the number of osteoclasts was significantly lower in the sham-operated PLCγ2^−/−^ mice than in the wild-type ones (3·0 ± 0·1 vs. 1·4 ± 0·5 per mm, respectively; *P* = 0·0036; *n* = 3), the number of osteoclasts was strongly increased and reached a comparable, though, still significantly different level (6·3 ± 0·4 vs. 5·0 ± 0·4 per mm, respectively; *P* = 0·010; *n* = 3) in the two genotypes after the ovariectomy procedure (Fig. 6e). The difference of the effect of ovariectomy on wild-type and PLCγ2^−/−^ animals (interaction of the genotypes and surgical procedures) did not prove to be statistically significant (*P* = 0·56; *n* = 3), indicating that PLCγ2^−/−^ animals were able to upregulate osteoclast numbers upon oestrogen deficiency normally. Analysis of the number of osteoblasts did not find any significant difference between any of the groups tested (Fig. 6e).

Taken together, these results suggest that ovariectomized PLCγ2^−/−^ animals are capable of reducing their bone mass to levels comparable to those seen in similarly treated wild-type animals, likely because of similar oestrogen deficiency–induced increase in osteoclast numbers in the two genotypes.

## Discussion

In the first part of this study, we showed that PLCγ2^−/−^ mice have increased basal bone density, likely due to reduced *in vivo* osteoclast number reflecting the role of PLCγ2 in a later phase of osteoclast development. These results raised the possibility that PLCγ2 may also participate in pathological bone resorption, such as oestrogen deficiency–induced osteoporosis. Much to our surprise, however, PLCγ2^−/−^ mice showed similar, or even more pronounced, ovariectomy-induced bone resorption than their wild-type counterparts. Therefore, PLCγ2 does not appear to be required for oestrogen deficiency–induced bone loss.

The experiments presented in this paper (part of which were published in an abstract form before [40]) were initiated based on our prior experiments showing defective osteoclast development and *in vivo* bone resorption in mice lacking immunoreceptor signalling adapter molecules or the Syk tyrosine kinase [10], as well as the similarity between various Syk^−/−^ and PLCγ2^−/−^ phenotypes [15,19,23–26,41]. However, two other groups have also independently reported *in vitro* osteoclast development and *in vivo* bone resorption defects in PLCγ2^−/−^ mice [42,43]. Although all three reports conclude that PLCγ2 is required for *in vitro* osteoclast development and basal bone resorption *in vivo*, they provide different explanations for those observations. Mao *et al.* [42] and Chen *et al.* [43] reported dramatically reduced expression of osteoclast-specific genes (such as those encoding TRAP, NFATc1, cathepsin K or the calcitonin receptor) in PLCγ2^−/−^ cultures, suggesting that PLCγ2 is required for an early step of osteoclast differentiation. A plausible explanation was that a PLCγ2-induced intracellular Ca^2+^ signal triggered activation of NFATc1, a Ca^2+^-sensitive master regulator of osteoclast-specific gene expression [31]. Surprisingly, our own more detailed analyses did not reveal any substantial defect of osteoclast-specific gene expression in PLCγ2^−/−^ cultures, and the expression of NFATc1 was not at all affected by the PLCγ2 mutation (Fig. 4b). Based on the time course of the expression of those genes and their low expression in parallel macrophage samples (Fig. 4b), it is unlikely that our results are attributed to non-specific amplification artefacts. It is also unlikely that our results are owing to inappropriate selection of the cytokine concentrations used because we did not observe reduced gene expression levels in PLCγ2^−/−^ cultures even when the concentration of both M-CSF and RANKL was reduced to 20 ng/mL, whereas the PLCγ2^−/−^ mutation caused severe osteoclast developmental defect even when the concentration of both cytokines was increased to 100 ng/mL (not shown). In addition, we consistently observed a large percentage of TRAP-positive mononuclear cells in PLCγ2^−/−^ osteoclast cultures (Fig. 2c), and such cells were also present in the PLCγ2^−/−^ osteoclast cultures shown by Mao *et al.* [42] and Chen *et al.* [43]. Taken together, osteoclast-specific gene expression is not (or not completely) blocked in PLCγ2^−/−^ cultures, necessitating alternative explanations for the PLCγ2^−/−^ osteoclast phenotype.

Another possible explanation for the PLCγ2^−/−^ osteoclast phenotype could be the participation of PLCγ2 in preosteoclast fusion, a process mediated in part by the DC-STAMP molecule [37,38]. While our gene expression studies (Fig. 4b) did not reveal any major role for PLCγ2 in RANKL-induced upregulation of DC-STAMP, it has yet to be tested whether PLCγ2 is involved in signal transduction by DC-STAMP or another preosteoclast fusion receptor.

Osteoclast development requires a complex interplay between signals from M-CSF, RANKL and ligation of adhesion receptors [44]. Because RANKL stimulation of adherent bone marrow-derived macrophages triggered PLCγ2 phosphorylation, Mao *et al.* [42] and Chen *et al.* [43] suggested that PLCγ2 is activated downstream of RANK. That conclusion, however, is confounded by the ligation of both RANK and adhesion receptors in that assay. In contrast, we did not observe any PLCγ2 phosphorylation upon stimulating wild-type macrophages with RANKL (or M-CSF) in suspension, whereas cellular adhesion consistently triggered robust phosphorylation of the protein in the absence of any cytokines (Fig. 5b,c). These results suggest that PLCγ2 is primarily involved in adhesion receptor rather than in RANK signal transduction, a possibility consistent with the role of PLCγ2 in integrin signalling of neutrophils [24,26] and its proposed modulatory effect on integrin signalling in preosteoclasts [45].

The analysis of ovariectomy-induced bone resorption is likely the most clinically relevant aspect of our study. While the increased basal bone volume (Fig. 1) and the defective *in vitro* osteoclast development and function (Figs 2 and 3) in PLCγ2^−/−^ mice suggested a role for PLCγ2 in pathological bone resorption, ovariectomy-induced bone loss in PLCγ2^−/−^ mice was unexpectedly normal or even more pronounced than in wild-type animals (Fig. 6). Because this was observed both in the distal femur and in the proximal tibia and by two independent approaches (micro-CT and histomorphometry), we believe that PLCγ2 is not a major general component of oestrogen deficiency–induced bone resorption. However, we cannot exclude the possibility that ovariectomy-induced bone resorption at certain sites or under some specific conditions would be defective in PLCγ2^−/−^ animals.

It is at present unclear to us how exactly PLCγ2^−/−^ animals are able to reduce their bone mass during oestrogen deficiency. However, the fact that the ovariectomy-induced increase in the number of osteoclasts was similar in the two genotypes despite significantly reduced basal number of osteoclasts in PLCγ2^−/−^ mice (Fig. 6e) suggests the existence of PLCγ2-independent mechanisms triggering oestrogen deficiency–induced osteoclast development. Whether those are mediated by cell–cell interactions (e.g. with osteoblasts) not present in the *in vitro* cultures [10,11], by excessive release of cytokines overcoming osteoclast developmental defects [46] or by the amplification of PLCγ2-independent signal transduction pathways, should be the subject of future research.

It has been generally believed that osteoclasts use similar signal transduction pathways during basal and induced (e.g. oestrogen deficiency–induced) bone resorption. Therefore, it has been assumed that the identification of novel osteoclast signalling molecules may provide suitable targets for the therapeutic intervention in pathological bone loss, such as postmenopausal osteoporosis. Our results showing normal ovariectomy-induced bone loss in PLCγ2^−/−^ animals indicate that this may not be the case. Interestingly, a prior study showed that ovariectomy-induced bone loss in the highly osteopetrotic DAP12^−/−^FcRγ^−/−^ animals is comparable to, or even higher than, that in wild-type mice [47]. All these results suggest differential osteoclast signalling requirements for basal and oestrogen deficiency–induced bone resorption and indicate that care should be taken when extrapolating findings on basal bone resorption to pathological conditions. These results also indicate that limited clinical benefit can be expected from therapeutic targeting of PLCγ2 in postmenopausal osteoporosis.

## Address

Department of Physiology, Semmelweis University School of Medicine, 1094 Budapest, Hungary (Z. Kertész, D. Győri, Z. Jakus, A. Mócsai); Independent Section of Radiology, Semmelweis University, 1089 Budapest, Hungary (S. Körmendi, C. Dobó-Nagy); Department of Immunology, Medical and Health Science Center, University of Debrecen, 4032 Debrecen, Hungary (T. Fekete, K. Kis-Tóth, E. Rajnavölgyi); Department of Internal Medicine 3, University of Erlangen-Nuremberg, 91054 Erlangen, Germany (G. Schett).
